# Improving Inclusivity in Robotics Design: An Exploration of Methods for Upstream Co-Creation

**DOI:** 10.3389/frobt.2022.731006

**Published:** 2022-06-21

**Authors:** Stevienna de Saille, Eva Kipnis, Stephen Potter, David Cameron, Calum J. R. Webb, Peter Winter, Peter O’Neill, Richard Gold, Kate Halliwell, Lyuba Alboul, Andy J. Bell, Andrew Stratton, Jon McNamara

**Affiliations:** ^1^ Department of Sociological Studies, University of Sheffield, Sheffield, United Kingdom; ^2^ Management School, University of Sheffield, Sheffield, United Kingdom; ^3^ Centre for Assistive Technology and Connected Healthcare (CATCH), University of Sheffield, Sheffield, United Kingdom; ^4^ Information School, University of Sheffield, Sheffield, United Kingdom; ^5^ Department of Sociology, University of Bristol, Bristol, United Kingdom; ^6^ Department of Computing, Sheffield Hallam University, Sheffield, United Kingdom; ^7^ Independent Researcher, London, United Kingdom; ^8^ P4C, London, United Kingdom; ^9^ Department of Robotics, Sheffield Hallam University, Sheffield, United Kingdom; ^10^ Advanced Manufacturing Research Centre (AMRC), Rotherham, United Kingdom; ^11^ Department of Computer Science, University of Sheffield, Sheffield, United Kingdom; ^12^ IBM Global, Portsmouth, United Kingdom

**Keywords:** social aspects of robotics, co-creation and co-production, social robots, disabled people, care robot acceptance, user—centered design, design thinking, lego serious play

## Abstract

Disabled people are often involved in robotics research as potential users of technologies which address specific needs. However, their more generalised lived expertise is not usually included when planning the overall design trajectory of robots for health and social care purposes. This risks losing valuable insight into the lived experience of disabled people, and impinges on their right to be involved in the shaping of their future care. This project draws upon the expertise of an interdisciplinary team to explore methodologies for involving people with disabilities in the early design of care robots in a way that enables incorporation of their broader values, experiences and expectations. We developed a comparative set of focus group workshops using Community Philosophy, LEGO® Serious Play® and Design Thinking to explore how people with a range of different physical impairments used these techniques to envision a “useful robot”. The outputs were then workshopped with a group of roboticists and designers to explore how they interacted with the thematic map produced. Through this process, we aimed to understand how people living with disability think robots might improve their lives and consider new ways of bringing the fullness of lived experience into earlier stages of robot design. Secondary aims were to assess whether and how co-creative methodologies might produce actionable information for designers (or why not), and to deepen the exchange of social scientific and technical knowledge about feasible trajectories for robotics in health-social care. Our analysis indicated that using these methods in a sequential process of workshops with disabled people and incorporating engineers and other stakeholders at the Design Thinking stage could potentially produce technologically actionable results to inform follow-on proposals.

## 1 Introduction

The last 10 years have seen a massive drive towards increasing automation in what some refer to as the ‘health-social care (HSC) ecosystem’ (Van Aerschot and Parviainen, 2020; Furst et al., 2021), i.e. the multiple, overlapping systems within which social care needs that arise from health-related conditions are addressed. Beyond the obvious intersections between the medical and the social, the ecosystem model also allows for inclusion of adjacent policy fields which impact greatly on the provision of care services but are not usually accounted for in these discussions (c.f. spending budgets, immigration policy, industrial strategy). Within this ecosystem there is also an increasing emphasis on inclusive design approaches which support a conscious effort to recognise the diversity of user perspectives (Maibaum et al., 2021), and the need for designers to develop ‘empathetic thinking’ (Berlach and Chambers, 2011). However, the user needs to be filled are often determined by external pressures. Resting on the assumption that population aging will be coupled with a concurrent decrease in both state financial support and professional care staff numbers,^1^ it is claimed that automation of services will enable fewer workers to care for more people (Charlesworth and Johnson, 2018), with market analysts framing the aging population ‘problem’ as an attractive and lucrative opportunity for private investment (Grand View Research, 2020; Deloitte, 2021).^2^ Hence, systematic reviews in the literature show that (rehabilitative robotics notwithstanding) the field is largely dominated by research targeting elderly care (Broadbent et al., 2009; Lazar et al., 2016; Abdi et al., 2018; Alnajjar et al., 2019), particularly the elderly with dementia (Koh et al., 2021; Ong et al., 2021). Although some research has considered elderly people at home, most has taken place in institutional settings (Vandemeulebroucke et al., 2018). These reviews repeatedly note methodological problems which make these research projects difficult to evaluate or compare (Broekens et al., 2009; Bemelmans et al., 2012; Kachouie et al., 2014; Abdi et al., 2018; Pu et al., 2019) and much is considered to be low quality, relying on small samples and self-reported measures (Papadopoulos et al., 2020). Evidence of benefit also tends to rely on studies examining short-term contexts, for example deploying the Paro robot seal in care homes (Pfadenhauer and Dukat, 2015; Wang et al., 2021), or narrow scenarios such as fall prevention/detection (Fischinger et al., 2016).

Hence, the potential end-user of most studies tends to be frail, dependent, and very elderly, overlooking differences in the needs, values and expectations of active-elderly and non-elderly people who need assistance to live their desired lives, as well as very different levels of comfort with complex technology. Roughly half of present care-users—and therefore roughly half of those who may be offered some form of robotics as part of their care package—are still under 65 (Kaye et al., 2010) and may have entirely different lifestyles and aspirations. Even when young disabled end-users are involved in research to develop assistive technologies, these are often aimed at specific needs, such as rehabilitation of a particular physical function (cf. Lu et al., 2012; Almenara et al., 2017) or singular tasks such as feeding (Song et al., 2013), so that the fullness of their lived experience may be lost through concentration on a particular problem to be solved. Because there has been comparatively little research involving social care robots carried out with physically disabled adolescents and young adults (Robinson et al., 2019), there is a danger that the responses given by elderly participants will be assumed to also reflect the needs of other groups which may have completely different tastes, experiences, values and goals.

Finally, user expertise is generally not sought until after development funding is secured and initial design decisions have been made, often not until prototypes which can be tested have been built (Reich-Stiebert et al., 2020 p. 228). By this point, development trajectory is difficult to change. Consequently, there has been a generalised movement towards developing design methods which can incorporate the perspectives of end users and other stakeholders at earlier, ‘upstream’ stages in the innovation process, potentially early enough to shape the research itself. While terms describing these may appear interchangeable, they do reflect subtle differences in how end-users are positioned relative to other stakeholders and the roboticist/design (RD) team. **Responsible innovation** advocates ongoing engagement to balance the needs of end-users with those of other stakeholders, from early upstream throughout the life of the project (Stahl et al., 2014). In **participatory design**, users have input into the initial design process (Šabanović et al., 2015; Cooper et al., 2016; Eftring and Frennert, 2016; Lee et al., 2017; Fischer et al., 2020), but decision-making still largely rests with the RD team. **User-centred/led/driven design** (Lu et al., 2012; Mast et al., 2012; Almenara et al., 2017; Ármannsdóttir et al., 2020) allows the end-user to define and repeatedly test potential solution(s) throughout the life of the project (much as RRI envisions), but the RD team has generally predetermined the problem domain. **Co-creation or co-design** (Davis et al., 2016; Čaić et al., 2018) represents a further step, so that responsibility for decision-making is shared between users and RD (and often other critical stakeholders), from defining the problem itself, to defining solutions and project goals, to producing outcomes. These models are increasingly used in service (re)design, although not always in a manner robust enough to challenge the dynamics of power inherent in technological decision-making, or the exclusion of marginalised users (Farrington, 2016). **Inclusive by design** takes a somewhat different approach, calling for the design of *all* products and services to be as inclusive as possible for as many people as possible (Clarkson et al., 2007; Coleman et al., 2007; Waller et al., 2010; Persson et al., 2015), rather than designing for a particular user-group.

However, attempts to move user engagement further upstream are not unproblematic. Scenarios offered for discussion or narrative elicitation using photos may present options limited by the designers’ expectations, which may not reflect the user’s needs or desires, and there is a general tendency towards paternalism when dealing with older or disabled people (Compagna and Kohlbacher, 2015). A hands-on study by Bradwell et al. (2019), for example, compared the responses of assisted living residents to pet-like robots with roboticists’ expectations and found the residents had a strong preference for animal robots to be lifelike and able to respond to speech or obey commands, all qualities which were undervalued by the roboticists, who had imagined ‘the elderly’ as a relatively passive group.

Given the ethical and social issues involved in using machines to augment human-delivered care at scale, a comparative exploration of whether and how different methods of elicitation can capture the complexities of how end-users imagine a robot might fit into their lives would therefore seem timely, potentially broadening the ways user input is solicited at the problem definition stage. In this paper, we reflect on an initial attempt to address some of these issues through methodological innovation in upstream engagement. Our goals for this were two-fold: one, to understand whether and how people living with disability thought a robot could improve their lives, and two, to investigate the kind of information different methods might elicit and how this could be made more useful to RD at an early-design stage. A full thematic analysis of the outputs of the workshops is discussed in Kipnis et al. (2022); in this paper we concentrate on the methods used, examining each of the workshop structures in detail before exploring how these shaped the themes arising. We then discuss the efficacy of the different workshop methods for eliciting design-useful information, and the broader implications this might have for improving upstream engagement in the field of care robotics.

## 2 Materials and Methods

Interpretive methods used at the very earliest stage of a design process may help reveal the underlying expectations of users, while allowing engineers and designers better access to the full experience of living with disability. This is essential to address a core knowledge gap, as few of those developing care robotics are themselves disabled, while users may hold misconceptions about the current state of the field -- created in no small part by the depiction of walking, talking, dancing robots in films, on YouTube,^3^ and in news items that suggest their use in care is imminent.^4^ Consequently, user-driven scenarios tend to be technically unfeasible, while values-based information is difficult for engineers to translate into technological specifications. In what follows, we allude to three particular tensions which may be ameliorated by the more inclusive approaches discussed below:

1. Misalignment between the needs of disabled users as perceived by RD, and what robots can do as perceived by HSC stakeholders/users;
2. The unique challenges of engaging potential end-users far upstream when care commissioners, care users and RD teams have heterogenous (and sometimes conflicting) values, needs, and externally-mediated aspirations;
3. The political and economic incentives for robotics innovation to be seen as a solution to the present adult social care crisis, reinforcing an individualised, medical model of disability which frames users as something broken to be fixed (Joshi and Šabanović, 2017; Lillywhite and Wolbring, 2019).

The tensions above may be considered beyond the scope of engineering solutions, however, they outline that it is essential for RD to understand how their robots may be deployed in social contexts. An interdisciplinary approach to stakeholder/user integration in early-design decision-making is necessary not only to ensure that the robots designed will be safe, reliable, and trustworthy in the intimacy of care contexts, but to also try to avoid harmful unintended consequences, such as state withdrawal of funding for human-administered care services in favour of automation which does not adequately respond to user needs.

### 2.1 Research Design

Overall, our ‘Improving Inclusivity in Robotics Design’ (IIRD) research programme aims to explore the context of automation in the health-care ecosystem and through this build a deeper understanding of what disabled adults, both younger and older, might consider to be a useful robot. The programme itself was developed at an interdisciplinary robotics sandpit held at the University of Sheffield early in 2019. IIRD-Phase I (July 2019) consisted of an open discussion workshop involving social scientists, disability scholars/advocates and roboticists/designers, with the purpose of illuminating questions which would benefit from mutual exploration. From this we identified early-design user engagement as key to better design knowledge, however, there were concerns from the engineering side about the usability of qualitative data generated without a specific robot in mind.

To investigate how this might be improved, we drew from the participants and organisers of Phase I to develop a strongly interdisciplinary team for IIRD-Phase II (2020-21). This project consisted of three focus-group workshops with adult members of the public who self-identified as physically disabled, contrasting two exploratory social science methods (*Community Philosophy* and *LEGO® Serious Play®*) with *Design Thinking*, a standard industry tool for product development. In these we attempted to remain as far upstream as possible, allowing participants to question even the idea of using robots to deliver health-social care. In a final workshop, we presented the results of the focus groups in the form of a thematic map to a team of roboticists and designers linked to a different funded project, to better understand how they would engage with such information if attempting to use it to develop an early-design specification. Our overall aim was methodological innovation for co-creative upstream (i.e. pre-proposal) engagement; we did not actually expect a viable specification to emerge.

In keeping with the theme of inclusive engagement, the entire research team, including the facilitators for the user focus groups, collaborated on the proposal submitted for funding and contributed to the overall research strategy and workshop designs. The team also participated in full pilots of each of the focus group workshops to help refine the format and allow everyone to experience the different methods first-hand. Because only three members of the team (facilitator, disability advocate and co-facilitator providing technical support) would be present during the focus group workshops, the rest were invited to attend an online replay of the video recording, recording their observations via the chat window during playback and in discussion afterwards. Both the chats and a video recording of the discussion were preserved as an aide to the analysis, since they represented the full research team’s interdisciplinary variety of expertise, which included software and robotics development, medical ethics, sociology, human-robot interaction, design, marketing, and technology assessment.

### 2.2 Focus Group Workshops

The three focus groups consisted of a total of 20 people, recruited via an intermediary agency. During pre-recruitment screening, participants were asked to complete a short series of yes/no questions indicating whether they had vision impairment, hearing impairment, mental health conditions, learning difficulties, acquired brain injury, autism spectrum disorder and/or physical disabilities. They were then asked to describe their disability in their own words. Five people indicated degrees of blindness, but all had some vision; four indicated they were hearing impaired (BSL interpretation was offered, as well as closed captioning, but was not ultimately required.) Interestingly, no one considered these to be *physical* disabilities. Applicants with cognitive or mental health conditions were not included in the final sample as these were considered to present different use-cases and/or different ethical issues, requiring specialist knowledge which was beyond the capacity and experience of the research team. However, we see no impediment to using these methods with people with these conditions, given the right expertise and with appropriate ethical safeguards in place.

Because of the COVID-19 pandemic the project, originally envisioned as face-to-face, was ultimately carried out completely online, utilizing Zoom as the platform most familiar to the participants. Participants were therefore also asked if their vision and hearing was adequate to participate in an online video room (again relying on self-assessment), and whether they were comfortable typing and moving small objects or had someone to help them with this (for purposes of group allocation). Each selected candidate was then allocated to one of the three focus groups by a combination of stated preference, availability, the requirements of that workshop (for example, manipulating small objects in the case of LSP), and ensuring for each group as wide a variation in ages, ethnicity and conditions as possible and a reasonable gender balance. The ages of the twenty participants ranged from 26 to 74, with the vast majority under 50, and nine were female. Five identified as either Black or Asian and fifteen as white. Overall, all but two participants were living with adult-onset relapsing-remitting or progressive conditions, and this variance in day-to-day capacities was to emerge from each group as a major concern, regardless of the method employed. Further details may be found in Appendix 1.

While the exact methods differ, all three focus group workshops revolved around the same two general questions: “How might a robot improve your life?” and “What would be the qualities of a useful robot?” Each group was led by a skilled facilitator accredited in the respective methods to ensure effectiveness. The methods were chosen because each has already demonstrated effectiveness as a tool for exploring values and experiences in groups which do not share a knowledge base, were flexible enough to be adapted to the project’s specific needs, and represented different elicitation techniques (open discussion, guided storytelling, brainstorming). While the move online did require some reconfiguration of the initial workshop methods (a topic we will discuss in greater detail below), it also had the beneficial effect of allowing us to recruit nationally across the United Kingdom, rather than only locally as originally planned, and removed the need for participants to travel to an unfamiliar location, which would have required effort considerable enough for some to preclude participation. Ethical approval was obtained from the University of Sheffield, and re-affirmed after reconfiguring the project to run online.

Below, we give a general overview of each workshop methodology and the analytic techniques used, before moving on to discuss the RD group which aimed to evaluate their combined thematic output.

#### 2.2.1 Community Philosophy

CP draws from a growing movement in which voluntary groups engage in philosophical thinking and action. Heavily influenced by Matthew Lipman et al. (1980), the founder of Philosophy for Children (P4C), its purpose is to develop reasoning skills and potential actions by considering both one’s own ideas and those of others in a guided discussion which encourages “thinking” as a practical tool for engagement in community and cultural life. Supported by a facilitator, participants enter into deliberations in which they challenge assumptions and reasonings together, in the expectation that new meaning or significance will arise as a result (Kennedy and Kennedy, 2011). The topic for conversation is often decided by the participants, however, in this project a more formal structure was pre-determined by the facilitator in order to ensure the discussions remained within scope, exploring ideas, experiences and beliefs around the key concepts of “care” and “robots” as a means to arrive at a deeper understanding of what they valued most in care situations. Eight people participated in this 3 h workshop. Because the method involved mainly verbal interaction, it was considered suitable for people with visual or fine motor-skill impairments which might preclude participation in the other two groups.

The initial focus was on building a sense of community so that everyone felt equally valued and ground rules relating to respectful interaction were collaboratively agreed. The first activity was a game called “would you rather” in which participants were asked to indicate whether they would prefer to have a robot that was practical, kind, or could premeditate wants and needs a screen-shared. During the next activity the participants explored the nature of real or imagined professional care interactions, with reference to all five senses, with the facilitator recording their contributions on an electronic whiteboard so participants could see the discussion develop. Activity three used a Good Idea/Bad Idea strategy, dividing the group into pairs in breakout rooms (not recorded), where each participant had to argue for 2 minutes, uninterrupted, for or against the statement, ‘Humans should be able to replace human care provision with robotic care provision’.

After all standpoints had been discussed, the participants had the opportunity to indicate the extent of their agreement with the statement by placing a mark on a horizontal line on the whiteboard, representing the agree-disagree continuum. This allowed for more conditional, contextual examples than had been previously offered. At the end of these activities, time was given to reflect on whether they had changed their original opinion. The final activity required participants to list what they valued most in terms of care provision, to reflect on whether they would be prepared to be supported in living with their disability by a robot, and to voice anything they had not had the chance to say.

#### 2.2.2 LEGO® Serious Play®

LSP is a facilitated thinking, communication and problem-solving method which uses specialised sets of LEGO bricks to surface knowledge and build stories about intangible ideas, bringing them into the physical world through the use of metaphor and play (Kristiansen and Rasmussen, 2014). It incorporates elements of neuroscientific understanding about the link between hand and brain in creativity (Rasmussen, 2006) and draws on participatory research from the fields of business, organisational development, psychology and learning (Wheeler et al., 2020). While initially developed for corporate strategizing and product or service design activities (cf. Pichlis et al., 2017), it is increasingly being used as a tool for academic research and higher education teaching (cf. James, 2013; Peabody and Noyes, 2017; McLeod et al., 2018). In an LSP workshop participants are led through a series of bespoke questions or “challenges”, building his or her response into a three-dimensional model which serves as the basis for individual narrative elicitation. These stories then build into group discussion, deepening knowledge in an iterative series of steps. As a research tool LSP has specific value in that everyone is given equal time and full attention, and its narrative qualities make it particularly well-suited for understanding lived experience (McCusker, 2014). All LSP workshops start with skills building exercises to ensure the participants gain confidence in their ability to put the bricks together, use them as metaphors, and construct a narrative describing the meaning of the models they build. Challenge sequences follow the four-step LSP methodology: (i) a carefully crafted question is posed; (ii) participants build a model to represent their answer, then (iii) take turns telling the story of their model; (iv) a short group discussion extracts additional insights (Executive Discovery, 2002).

For this workshop, the challenges asked participants to build a model to:

1. represent a tower (basic skills building)

2. describe “a good day” (use of metaphor skills)

3. characterise an ideal experience of being cared for or supported (opening the topic)

4. describe what the words “easy” and “effortless” mean to them (as care robots should make life less difficult)

5. describe the characteristics which would make a robot suited to a task they found otherwise difficult to accomplish

6. describe a principle which robot designers should use when designing care robots (generating several small models).

This workshop consisted of six people and was carried out in one 3.5 h session. Participants were sent small, specialised kits of LEGO in advance and a MIRO electronic whiteboard was used as a tool for mediating challenge 6, using electronic post-it notes to represent the theme of each model so all could be seen at once.

#### 2.2.3 Design Thinking

The methods of Design Thinking emerged from the realisation that modern design can and should be something more than simply packaging an existing idea in a way that is attractive to consumers. Instead, designers should be involved in the creation of those ideas in the first place, using their own “sensibility and methods to match people’s needs with what is technologically feasible and what a viable business strategy can convert into a customer value and market opportunity” (Brown, 2008 p. 86). The DT process is typically thought of as involving two phases (Lindberg et al., 2011), each consisting of divergent and convergent activities, similar to the “double diamond” framework for innovation (McNabola et al., 2013). In the first phase, the conceptual space of the problem is explored, and in the second possible solutions are proposed (iteration between the two phases is possible). During exploration of the problem space, activities are undertaken to help designers understand users and the context of their problems through involving them in observational studies, interviews, scenario development or any other appropriate method of engagement. Exploration of the solution space consists first of an “ideating” stage, in which multiple ideas are generated in as free (divergent) a manner as possible so as to avoid obvious and conventional solutions, followed by a “prototyping” phase, in which artefacts (prototypes, mock-up, wireframes, etc.) are generated to help elaborate design concepts and, with user testing and feedback, eliminate weaker candidates until the focus falls upon one or two to develop further (convergence).

Within IBM, which was a partner to this part of the research, this model is often integrated with agile software development processes (Lucena et al., 2017), as a way of ensuring that user needs are incorporated early so that development efforts are expended on solving the correct problem. Particular tools that are used during the problem space exploration phase (here termed the “visioning phase”) include user personas, empathy maps and storyboards, while the solution space exploration (“delivery wave”) incorporates engineering concepts such as sprints and integrates feedback from users. In the context of this project, we were only interested in the visioning phase, that is, in the initial exploration and refinement of the problem to be solved by robotics. The techniques in this phase are relevant to any form of technology development, so could be productively adapted for this case.

The DT workshop was the most technologically demanding, requiring that the participants attend a pre-call the day before the workshop to become familiar with a bespoke whiteboard environment on the Mural platform, as they would have to interact directly with the board to fulfil the tasks. This was not recorded, as it was not part of the dataset. The actual workshop took place the next day over a period of 5 hours (including an hour for lunch) and was composed of six people, led by two facilitators from IBM. The workshop itself consisted of five sets of tasks, each of which involved a question, a period of silent ideation during which participants put ideas on the whiteboard in the form of post-it notes, then discussion of the notes and feedback. Between each task the facilitators rearranged the post-its based on the prior conversation, for clarity and to serve as the starting point for the next iteration.

Task 1 introduced the participants to a ready-made male user persona, “Jamie”, developed by the facilitators as an anonymised reflection of the participants’ range of disabilities, as self-reported on their application to the project. This task asked participants to explore Jamie’s morning routine, considering what he was doing, thinking and feeling as he prepared to leave the house for work, and what “sticking points” he might encounter along the way. Task 2 involved ideation of “big ideas” which might help Jamie overcome those points; these did not have to be logical or feasible. A series of votes then narrowed the choices down to five. Task 3 asked participants to then order these on a prioritization grid, looking for the most feasible solution with the highest impact. This formed the basis of Task 4, in which each participant constructed a story of Jamie starting his day using that solution. The facilitators then amalgamated all the stories into a single narrative. The final task examined the assumptions being made, and identified outstanding questions to be answered before the new technology could move to a prototyping phase.

### 2.3 Roboticists and Designers Workshop

The final workshop in the sequence served as both observational data collection (about the knowledge requirements and reasoning processes used in early engineering design decision-making), and as an analytic technique in its own right. Its purpose was to evaluate the quality of the information that had emerged from the focus groups, and determine whether this could have a constructive influence on the development of a (theoretical) prototype.

Unlike the other workshops, which adapted existing tools to our purpose, the novel nature of the RD workshop meant that no proven methodology existed, and, as a result, the research team had to invent its own approach. The workshop was facilitated by a team member with an academic background in engineering design. A second team member documented the activity through the use of an online Miro whiteboard; a third acted as a navigator for the thematic map, while the fourth, who had occupied a safeguarding role at the focus group workshops and is himself disabled, now acted as advocate for the participants, as he had not been involved in the analysis which produced the map. Together, these three formed a panel of “oracles” who could be questioned in detail about various aspects of the focus group methods and activities, participants, information elicited, or about the task the RD group had been set (although as far as possible it was left to that group to decide how to go about it). The general structure of the workshop was as follows:

1. Introduction to the aims of the project and a brief overview of the three focus group workshops and the characteristics of the participants.
2. Introduction to the structure and content of the thematic map generated through analysis of the workshops (participants were emailed this as a PDF they could browse independently).
3. Main task: to work together to develop a *valid specification* of a robotic system guided by the information in the thematic map or, if they felt that this was not possible, to explain why the information was inadequate and what further questions they felt they needed to ask.
4. Evaluatory discussion of the methods used, and feedback to the research team.

The format was first piloted with three people external to the project who are all involved in academic RD. The actual 2.5 h workshop consisted of six roboticists and designers (none of whom had taken part in the pilot), known to the research team as having the required level of understanding of robotic design. For our purposes, a *valid specification* was defined as a coherent description of a robot system consisting of the following six elements:

- A logic model, stating what benefits the robot would provide, for whom, in what manner, and under what conditions.
- Functionality requirement(s), achieved by a combination of structure and behaviour (or hardware and software).
- Interfaces, including user interfaces and any interfaces to other technical systems (such as the internet).
- Desired performance goals, such as response speed, or operational lifetime.
- Non-functional and/or systemic aspects, such as appearance, materials, and physical size or contextual requirements such as training needs, support and maintenance.
- Any additional operating constraints or assumptions. These could include the environment in which the robot would be used, or aspects such as availability of internet connectivity.

*Valid* was defined as a physical robotic device (rather than an app, chatbox or device whose robotic elements were trivial), not replicating an existing system, and feasible within the next 3–5 years given the state of the art. It had to satisfy at least one of the *functional requirements* identified in the thematic map, and as many of the other *qualities of a useful robot* as possible, but without adding arbitrary complexity or cost.

To encourage analytic thinking, participants were asked to verbalise why they were focusing on certain information and any implications drawn or decisions made on that basis, and to identify any aspects of the thematic map they felt to be inadequate, missing, or otherwise required to assist the design process. Ideas were simultaneously recorded on a (not screen-shared) Miro whiteboard which was divided into the six elements. Once the time allocated for the task was complete, the whiteboard was shared and the design team was asked to reflect on its contents, and to consider whether or not their specification was complete, consistent, and met the given validity criteria. They were permitted to revise the contents as necessary to arrive at a more satisfactory specification. Following this, the oracles were then invited to consider this revised specification, and request clarification of any aspects that were vague or ambiguous.

The group as a whole was then asked to jointly consider the value of the specification in terms of its novelty, feasibility, potential benefits and possible cost-effectiveness, and whether there would be any merit in developing such a specification further. Finally, the group was asked to reflect on the design activity and to compare it with other design processes they had been involved with, specifically to consider whether the informational content felt qualitatively different, and any ways in which the activity might have been improved.

### 2.4 Analytic Techniques

All workshops were audio and video recorded with participants’ consent, simultaneously captured in three modes: speaker (particularly important to see the models in LSP), gallery (allowing us to observe interactions between participants) and screenshare with speaker (to capture activity which took place outside the Zoom environment using the electronic whiteboards). Transcripts of the focus group audio data were then subjected to analysis by a subgroup of three researchers using two distinct methods: thematic analysis and argumentation mapping, with the video data providing verification of speaker and enabling matching with the whiteboard outputs. Although disparate techniques, the combination allowed for an examination of both the argumentation trajectory of each workshop and its discursive content, to enable a deeper understanding of what kind(s) of knowledge and information each method was able to elicit from participants.

#### 2.4.1 Thematic Mapping

Because we wanted to explore what each methodology offered, we chose not to predetermine a theoretical structure, instead analysing the themes as they arose from the discussions through a process of constant comparison and refinement (Glaser, 2002) which could emphasise the grounded, situated nature of the participants’ individual knowledge and experience (Gioia et al., 2013). We were aware that to some extent, the conversations would be shaped by the method used, but did not necessarily know how, and this is part of what we set out to discover. We also could not expect to achieve the categorial saturation typical of grounded methodologies, as this would not be possible with such a small sample. Following this reasoning, the thematic analysis strategy employed a systematic inductive approach developed by Gioia and others (Corley and Gioia, 2004; Gioia et al., 2013), which is well established in consumer and service research (cf. Sharma and Conduit, 2016). This recommends structuring the data to inform analyses and interpretation as follows:

- Participants’ views and expressions are organised as *first-order concepts.*
- *Second order themes* are subsequently derived as an analytical interpretation of commonality amongst the first-order concepts.
- These are then aggregated into key *analytic dimensions* capturing the broad focal areas of the workshops.

**FIGURE 1:**
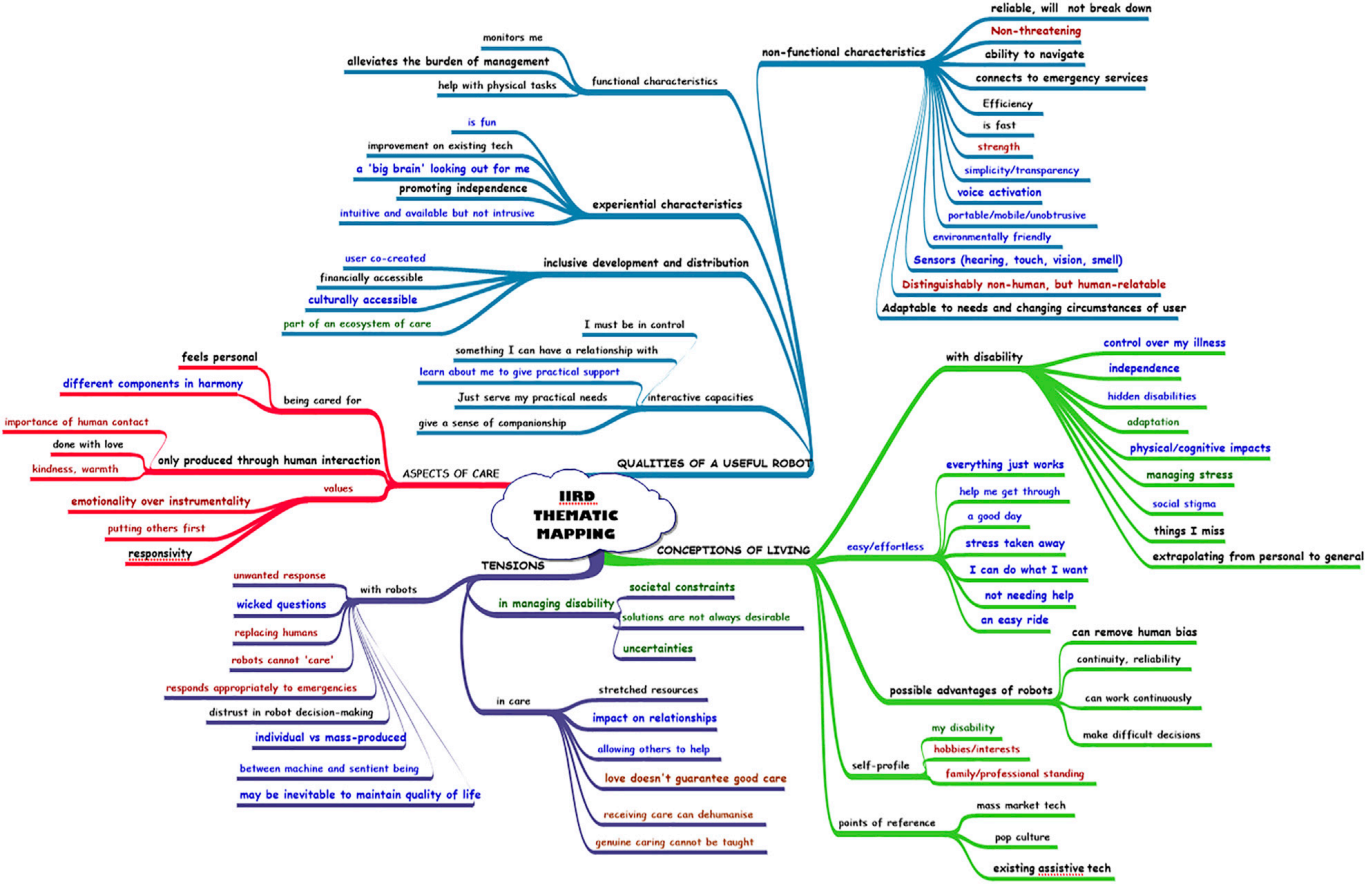
IIRD Thematic Map.

Two members of the research team coded the data of each workshop independently. Subsequently the coders compared first order codes and second order themes emergent from their analyses, reconciling and consolidating their interpretations. As an additional check for robustness, the final data structure for each workshop was contrasted against the mapping of argumentation conducted independently by a third coder (discussed below), who also acted as arbiteur during the reconciliation phase of the thematic analysis process. The emerging data structure was then recorded utilising Inspiration 9.2,^5^ a mindmapping tool which produces a visual map of the thematic structure, with analytic dimensions branching into second order themes, divided into first order concepts linked to the transcript text. In the final step, the maps from each workshop were amalgamated to produce a single thematic map for use in the RD workshop (Figure 1). In this map, each group has been assigned a specific colour: CP red, LSP blue and DT green. Where the text for the first and second order themes display one of these colours, it means that theme arose only in that group. Themes which arose in more than one group are coloured black. Colour of the branches is not relevant. Specific themes and the workshops in which they appeared may be found in Tables 1–4.

**TABLE 1:** Conceptions of living.

Aggregate Dimension	2nd Order Theme	1st Order Theme	CP	LSP	DT
**CONCEPTIONS OF LIVING**	**with disability**	Control over my illness	—	**X**	—
		Physical/cognitive impacts	—	**X**	—
		Adaptation	—	—	**X**
		Managing stress	—	—	**X**
		Things I miss	—	**X**	**X**
		From personal to general	**X**	**X**	**X**
		Social stigma	—	**X**	—
		Hidden disabilities	—	**X**	—
		Independence	—	**X**	—
	**points of reference**	Mass market tech	**X**	**X**	**X**
		Pop culture	**X**	**X**	**X**
		Existing assistive tech	**X**	**X**	**X**
	**easy/effortless**	Everything just works	—	**X**	—
		Help me get through	—	**X**	—
		A good day	—	**X**	—
		Stress taken away	—	**X**	—
		I can do what I want	—	**X**	—
		Not needing help	—	**X**	—
		An easy ride	—	**X**	—
	**possible advantages of robots**	Can remove human bias	**X**	—	—
		Continuity, reliability	**X**	—	—
		Can work continuously	**X**	—	—
		Make difficult decisions	**X**	—	—

**TABLE 2:** Aspects of care.

Aggregate Dimension	2nd Order Theme	1st Order Theme	CP	LSP	DT
**ASPECTS OF CARE**	**Values**	Responsivity	**X**	—	—
		Putting others first	**X**	—	—
		Emotionality over instrumentality	**X**	—	—
	**being cared for**	Feels personal	**X**	**X**	—
		Different components in harmony	—	**X**	—
	**only produced through human interaction**	Importance of human contact	**X**	—	—
		Kindness, warmth	**X**	—	—
		Done with love	**X**	**X**	—

**TABLE 3:** Qualities of a useful robot.

Aggregate Dimension	2nd Order Theme	1st Order Theme	CP	LSP	DT
**QUALITIES OF A USEFUL ROBOT**	**functional characteristics**	Monitors me	—	**X**	**X**
		Alleviates the burden of management	—	**X**	**X**
		Help with specific physical tasks	**X**	**X**	**X**
	**experiential characteristics**	Intuitive but not intrusive	—	**X**	—
		a “big brain” looking out for me	—	**X**	—
		Is fun	—	**X**	—
		Improvement on existing tech	—	—	**X**
		Promoting independence	**X**	**X**	—
	**non-functional characteristics**	Environmentally friendly	—	**X**	—
		Simplicity/transparency	—	**X**	—
		Portable/mobile/unobtrusive	—	**X**	—
		Voice activated	—	**X**	—
		All senses	—	**X**	—
		Ability to navigate	**X**	**X**	—
		Strength	**X**	—	—
		Non-threatening	**X**	—	—
		Non-human but human-relatable	**X**	—	—
		Reliable, will not break down	**X**	**X**	—
		Adaptable to changing needs and circumstances of user	**X**	**X**	—
		Connects to emergency services	—	**X**	—
		Efficient	**X**	—	—
		Is fast	—	**X**	—
	**interactive capacities**	Learn about me to give practical support	**X**	**X**	—
		Gives a sense of companionship	**X**	—	—
		I must be in control	**X**	**X**	—
		Just serve my practical needs	**X**	**X**	—
		Something I can have a relationship with	—	**X**	—
	**inclusive development and distribution**	Part of an ecosystem of care	—	—	**X**
		User co-created	—	**X**	
		Financially accessible	—	**X**	**X**
		Culturally accessible	—	**X**	

**TABLE 4:** Tensions.

Aggregate dimension	2nd order theme	1st order theme	CP	LSP	DT
**TENSIONS**	with robots	Unwanted response	**X**	—	—
		Wicked questions	—	**X**	—
		Replacing humans	**X**	—	—
		Robots cannot “care”	**X**	—	—
		Responds appropriately to emergencies	**X**	—	—
		Distrust in robotic decision-making	**X**	—	**X**
		Individual vs mass-produced	—	**X**	—
		Between machine and sentient being	—	**X**	—
		May be inevitable to maintain quality of life	—	**X**	—
	in managing disability	Societal constraints	—	—	**X**
		Solutions are not always desirable	—	—	**X**
		Uncertainties	—	—	**X**
	in care	Stretched resources	**X**	—	**X**
		Impact on relationships	—	**X**	—
		Allowing others to help	—	**X**	—
		Love doesn’t guarantee good care	**X**	—	—
		Receiving care can dehumanise	**X**	—	—
		Genuine caring cannot be taught	**X**	—	—

#### 2.4.2 Mapping of Argumentation

To provide a complementary, goal-directed approach, we also analysed the video recordings using the constructs of the Issue-Based Information System (IBIS), an argumentation-based approach for clarifying and, to a certain extent, formalising collaborative problem-solving activities (Conklin and Begeman, 1988; Kunz and Rittel, 2010). The use of IBIS to capture design rationale relies on the insight that (co-)design, especially in its early stages, is a process of argumentation (Noble and Rittel, 1988). While normally done *in situ*, as part of the design process, we suggest that an IBIS-based analysis can be performed retrospectively on a video recording of the discourse to 1) clarify the structure and outcomes of the discussion, including any unresolved matters; and 2) provide a basis for a comparative evaluation of different collaborative design methodologies.

The IBIS model consists of the following constructs or *nodes*:

- *Issue*: typically in the form of a question that needs to be answered. This might concern the task at hand, the design process itself, or even the wider context in which it is taking place.
- *Position*: a possible idea (answer) to resolve the issue (question).
- *Pro*: an argument in favour of a position.
- *Con*: an argument against a position.

We also adopted two additional nodes which have been incorporated into the Compendium mapping tool (Buckingham Shum et al., 2006),^6^ a software implementation of the IBIS approach which was used to create the maps:

- *Decision*: to indicate some agreed outcome or result of the process
- *Note*: the provision of information outside any specific argument.

The archetypal model of argumentation adopted for IBIS is that someone in the group raises an issue to which one or more people will respond with one or more positions. In turn, each position gives rise to pros and cons. However, an issue might also give rise to another issue, or be suggested by a position, or by one of the pro or con arguments, and so on. Each of these nodes is documented as it arises, represented according to its type (issue, position, etc.) with a textual label summarising its contribution. It is also connected with one or more arrows pointing to the node(s) which gave rise to it. In this manner, a directed graph or “map” of nodes and arcs (arrows) is constructed, diagrammatically representing both the structure of the argument and its content.

The IBIS approach is normally used for “live” mapping of argumentation, during which the emerging map is visible to all participants. Used in this way it serves both as a structured means of documenting the discussion, but also, more subtly, as a way of controlling and directing the discourse (albeit in a fairly loose fashion) by suggesting the form of subsequent valid moves in the argumentation “game”. While the focus groups did not seek an agreed outcome, mapping the discussion retrospectively did produce insight about the nature of the method in question, and the kind of argumentation possibilities it produced.

#### 2.4.3 Content Analysis of RD Workshop

In addition to IBIS mapping of the core activity of the RD workshop, examining this session’s transcript using quantitative content (Stemler, 2000) allowed us to count instances when the RD team referred to the IIRD thematic map, offering some insight into the design-relevance of those elements. To do this, themes from the map were located in the RD transcript through keyword searches and unique references to these were recorded. Theme keywords were generated using a conceptual thesaurus (Carley, 1997) in the text processing program Automap^7^ to aggregate words from the thematic map (e.g., *Robots* becomes *Robot*). The same process was applied to the RD transcript to ensure alignment between the written and conversational use of language (e.g., changing *tech* to *technology*). Of the 223 unique keywords generated, 143 also appeared in the transcript. Of these, the nouns, verbs, adjectives, and adverbs were identified as candidate keywords to search through the transcript; high-frequency ‘noise’ keywords, such as *the, and, be* were excluded, leaving 104 keywords for the search. See Appendix 2 for the list of terms aggregated using stemming and the list of keywords drawn from the IIRD map. While content analysis in the form of word frequency has limitations based on the varying contexts in which the keywords may appear, inspection of the transcript was used to confirm relevant usage (eg. Stemler, 2000).

## 3 Results

The completed thematic data structure showed second order themes aligning in four key aggregate dimensions: Conceptions of Living, Aspects of Care, Qualities of a Useful Robot, and Tensions (where participants either indicated major concerns or two opposing perspectives on the same topic emerged). Some second order themes emerged in all three workshops; others were specific to one or two in different combinations. In what follows we present the trajectory of each workshop and how the thematic findings were enabled by the methodology used, before discussing the argumentation structure. Subsequently, we present our evaluation of the usefulness of each method we deployed through examining the RD group’s interaction with the combined thematic map.

### 3.1 Community Philosophy

The majority of themes within the dimension *Aspects of Care* were generated within the CP workshop, reflecting the participants’ primary concern, which centred on the inability of machines to actually “care” in the emotive, compassionate sense of the word. Although certain themes in this dimension also arose spontaneously in the LSP group, the majority originated as responses to an extended exploration of how it felt to be cared *for,* which they considered to be a reciprocal act of “*giving and receiving*” (CP/D), putting others first, being responsive and acting with kindness. Overall, there appeared to be a consensus this could only be produced through human interaction. As one participant put it:

“*I think kindness stems from inside a human being towards another human being. And I think, without being rude, I think expecting kindness from a robot is probably like expecting love from a blow-up doll.*” (CP/R)

Crucial distinctions were also made between the emotional and instrumental aspects of care, between the *what* and the *how*:

*“That act of wiping my face doesn’t necessarily mean that you care about me. It’s an act. So it’s that genuineness of the compassion, that somebody is doing that so softly or gently or meaningfully, that comes across in the warmth, in the emotions of that individual. Rather than it being ‘I’m just wiping down a surface’.”* (CP/Lo).

The Good Idea/Bad Idea strategy also revealed key ambiguities in the automation of care, precisely because robots have no emotions. These mainly informed the dimension *Qualities of a Useful Robot* and included improved access to care (e.g., robots would not get ill or tired, they could work continuously and thus take some of the strain off the NHS) and a more consistent quality of care (e.g., avoiding errors caused by omission or bias, being better at diagnosis). However, the lack of emotion pointed to an inherent ambivalence if robots were used to make ethically or emotionally fraught decisions:

*“Carrying on care, for example, when there was very limited reason to do so, but a human might make an emotional decision to carry on […] a robot would go, ‘well, this is going to cost £60,000 to keep this person alive for five weeks and that’s not efficient.’ And that’s brutal, isn’t it? […] I wouldn’t want them to make that decision for my dad. But you know, I think as a greater good thing, it does help us get a bit more potentially efficient …* ” (CP/S)

In listing what they valued most in terms of care provision, though they seemed to value human qualities (such as compassion, consideration and empathy) very highly, participants also expressed their values in terms of design principles for roboticists (eg, natural conversation and user-friendly appearance). Some participants did find it acceptable that a robot was only able serve practical needs, “*transactional things that a robot would be able to do for me, which are quite helpful and I would not necessarily call it like in terms of care*” (CP/Ly). Overall, however, the participants reasoned that ‘being cared for’ is a multifaceted experience which robots would never be able to actually emulate:

*“…the empathy/ sympathy side wouldn’t be there. Because it’s the emotional attachment that you have with a human that you wouldn’t necessarily have with the AI. […] the humour and the comedy element of a human, you just would not get that with a robot unless you had to programme it and then it wouldn’t be natural.” (CP/E)*

In this sense “care” was attached not only to empathy or compassion, but to a particular kind of person, one who could respond spontaneously to make the person feel cared *for,* although it was also argued that functions such as polite speech could be built into the software design to produce an acceptable simulacrum of empathic feeling. Overall, the more values-based structure of this workshop lent itself best to a close examination of the subtle differences between *being* cared for and *feeling* cared for, suggesting that regardless of the task a robot might actually perform, without the latter the former could not be achieved.

### 3.2 LEGO Serious Play

In contrast to CP, the storytelling aspect of LSP provoked a personal, contextualised response which allowed diametrically opposed experiences to remain side by side without a need to reconcile them or attempt to see the other point of view. From an analytic perspective, this can usefully demonstrate the range of possible responses even within a small sample. For example, one participant defined being cared for as feeling like “*the king of the castle … I’m the centre of focus … pampered to an extent*” (LSP/S), an experience he found to be a positive aspect of being ill, while another defined it as “*the ability to allow me to carry on caring for myself*” (LSP/Da).

On the thematic map, the second order code “easy/effortless” appears only here as this derived from a specific challenge asking participants what it meant to them. Most of the themes LSP generated fell into the dimensions of *Qualities of a Useful Robot* and *Conceptions of Living*, a dimension which did not generate much depth in the discussions in CP. Both the warm-up challenge about a good day and the challenge to describe easy/effortless prompted a similar range of responses, along the lines of “everything just works” (LSP/A). Asking specifically about tasks a robot could perform, however, contributed to a fuller discussion of living with their particular disability and its impact on their identity. As with CP, since most of the users in this group were living with episodic and/or progressive conditions, management of variability emerged as a key concern. Most of the robots envisioned had some kind of quasi-autonomous embodied AI able to ‘look out’ for the user in a number of ways, such as:

*“…helping with the mental side of things, like the organisation and the worrying and the stress, and trying to sort everything out, and remembering to, like, order your meds and stuff. …basically my robot’s got a big brain to help me organise everything and work things out.”* (LSP/A)

**FIGURE 2:**
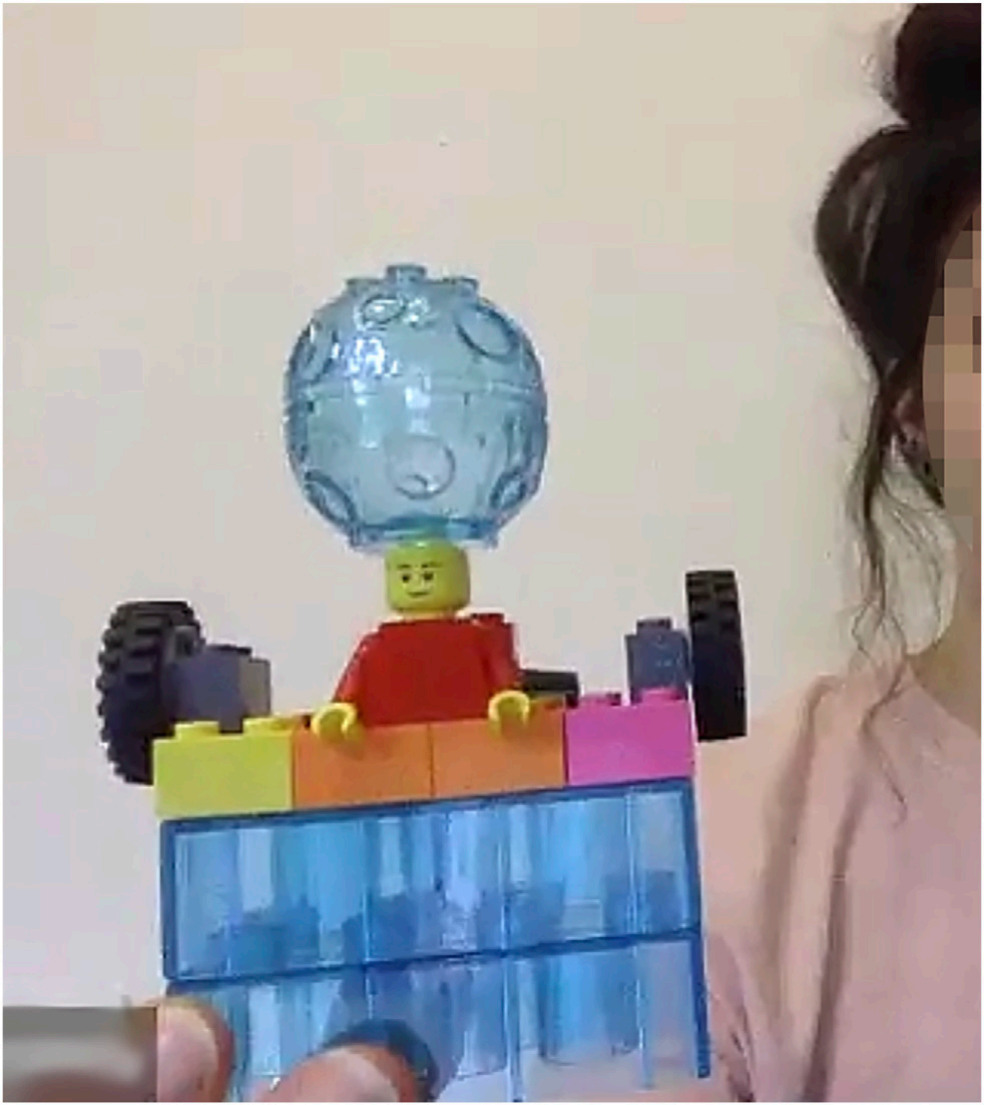
The “big brain”.

Once introduced, this metaphor of AI as a ‘big brain’ (see Figure 2) was then picked up by others in the group to describe similar management functions:

*“But the important thing for this big brain on top, or this antenna, is actually monitoring all the vital symptoms, because that’s my problem with my disability, that I can have a cardiac arrest at any moment, and actions have to be taken quite quickly …* ” (LSP/S)

Because the second main question focused on tasks, this also included ways a robot could learn about the user’s actual needs to increase support (as opposed to supporting bad habits which could be injurious), for example, to *“keep pushing me along as much as I need to do, but at the same time, I’ve got that lasso so he can rein me in when I’m going too far”* (LSP/Dm), or to *“know what you need even before you know it, sometimes”* (LSP/A). For both groups, however, being in control was paramount. This was best exemplified by a participant in the LSP group who expressed a desire to use the robot to help him maintain control in the face of a progressive illness, which went hand-in-hand with control of the robot itself:

“*I think with my own condition, because it is progressive, I’ve found you reach certain points where you cross into another part of the disability, and looking at the AI that would go with this, and the robotics of it, to step into that world and then progress through that world, I want to be in control of [the robot], not somebody else remotely ... I want to be in control of my own illness, and it is as simple as that* (LSP/Da).

Although fewer themes emerged in the *Aspects of Care* dimension during the LSP exercises, those which did exhibited a similar understanding of the humanistic nature of “care” as in CP:

*“I feel that care and consideration, no matter who you’re caring for, requires love to be able to do the job properly, whether that’s love of your job or love of the person you’re caring for”* (LSP/Dm).

However, the LSP group did also generate a theme of robots as ‘something I can have a relationship with’, a quality that was desirable to some, particularly if there was an expectation of increasing dependency:

*“…it’s actually monitoring me, but it’s still fun. I actually believe that there’s a relationship with my robot, that it's got artificial intelligence, and it learns about me and adapts. … It is like a person to me. I would spend a lot of time with it. … it doesn’t dominate my life, but it’s an integral part of it, and I want to be happy with it.”* (LSP/S)

The majority of first-order concepts in the *Qualities of a Useful Robot* dimension were generated from the final challenge in the LSP workshop, where participants were asked to create a number of small models representing essential principles of a useful robot. Although having begun with very personalised accounts of what a robot could specifically do for them, this prompted a shift to more universal considerations, such as affordability, ease of use, and in particular the ability to adapt to a user with changing needs. Without being introduced to the idea of co-creation, the possibility of an ongoing group of people with disabilities helping to develop the robots they would one day use arose spontaneously and was roundly approved:

“I would definitely agree with the user group [working with designers] throughout the whole process … there’s so much that goes on behind the scenes [of living with disability], like so many tiny things that you have to deal with or find workarounds for in everyday life, that other people just would never think of.” (LSP/A)

Overall, because the outlook was generally positive, the *Tensions* outlined by the LSP group tended towards the systemic, in particular tension between individual needs and likely mass production, and the possibility that even if unwanted, some form of robotic care might be the only way of maintaining a good quality of life.

### 3.3 Design Thinking

*Tensions* under the second-order theme “in managing disability” emerged mainly from the DT workshop, which also contributed significantly to *Conceptions of living* “with disability”, perhaps owing to its focus on the specific daily tasks involved in getting “Jamie” ready for his day, and trying to navigate life within current systems of social organisation. The topic opened up a discussion of the participant’s varying conditions and the obstacles they themselves faced on a day-to day basis (in particular the increasing lack of public toilets, the need for which could be sudden and urgent). While their disabilities and therefore their “sticking points” varied, as with LSP most in this group had chronic, variable conditions that participants often struggled to manage. These factors were identified as creating additional pressures beyond the condition itself:

*“Everyone’s got their own disabilities, but it affects most of us, we’ve all got an idea of the stress around getting ready, getting up early, that kind of thing, toilet facilities. Everyone’s got similar kinds of things.”* (DT/R)

*“...a lot of stress and anxiety is knowing something’s going to happen, but not being able to predict [it]… I suppose for me, it’s like if you know you’re going to be sick, the feeling of knowing you’re going to be sick is actually worse than being sick itself.”* (DT/W)

The sequence of ideation and prioritization activities in the first half of the workshop, therefore, quickly homed in on a desire for a kind of multi-functional app or suite of apps which could monitor vitals and give predictive management advice, connect to GP or ambulance services if necessary, or even locate accessible toilets, tasks for which it was not clear an actual robot would be needed.

The second half was given over to each participant imagining a scenario in which an app suite would be used to get Jamie to work on time. Although there was some variation here, the overall trajectory was that Jamie had a need, eg. an unidentified pain or blood sugar fluctuation, or had to provide a daily measurement, and was able to use the app to investigate that need (potentially using additional tools such as a blood pressure monitor) and determine what steps to take next:

*“And basically, in turn, that would identify any issues and diagnose you with potential conditions, and then give you a report maybe. And if there’s anything severe, you could get fast tracked through the NHS system, and have a doctor get in touch with you rather than the other way round.”* (DT/M)

The separate scenarios were then amagamated into one narrative by the facilitators to collate all the salient points.

In terms of our exploration, the structure of the DT workshop meant it generated less themes overall as much of the reasoning process takes place in silence, and much of the discussion consisted of facilitator interpretation of the developing solution. Therefore, despite this being the longest workshop, once the facilitators’ speech was filtered out there was significantly less text to analyze and, apart from the initial task exploring participants’ own experiences of starting their days, the imagined scenarios of usefulness were more homogenous. This strongly suggested that the highly structured visioning phase of DT was perhaps most useful in synthesis of different experiences, rather than the kinds of open exploration enabled by CP and LSP.

### 3.4 Mapping the Different Methodological Processes

Since the format, methodology, content and duration of the workshops differed considerably, we can draw few conclusions from a direct comparison of the contents of the resulting IBIS argumentation maps. The use of silent ideation in the DT workshop in particular, means that it was less susceptible to this sort of analysis—in some sense, the very methodology of DT is its own argumentation structure as it is meant to lead to one or more design decisions. Nevertheless, we can make three general observations about the argumentation deployed in the workshops as revealed by the IBIS-based analysis:

- All the workshops displayed a relatively ‘shallow’ argumentation structure: an issue (question) would typically be addressed by one or more positions (answers), which would rarely lead to extended ‘deep’ structures raising further questions, and then more potential answers, and so on. This was particularly noticeable in the LSP workshop as the particular protocol used was not intended to lead to a negotiated outcome.
- Issues–which typically form the starting point for argumentation–were almost always raised by the facilitators of the workshop, while participants supplied positions and pro/con argumentation for those positions.
- Issues were therefore almost always left ‘unresolved’ in the sense that there was no attempt to seek a clear decision made among “competing” positions/answers. Instead, the issue was typically considered “closed” when no further positions were suggested by participants–either when saturation/exhaustion of ideas/opinions had been reached or it was felt that everyone had “had their say”.

Taken together, these shared characteristics would seem to reflect their use in this context; it is compatible with the idea of an initial exploratory phase of design in which the problem space is explored and expanded. (It is worth noting that the problem space here includes elements which might be considered to properly belong to the solution space, since it is difficult to talk about the problems that might be solved by robotic systems without talking, in some manner, about robots.) Had the participants also included designers or roboticists, there might well have been more questions raised by the group itself (rather than the facilitators), and passages of “deeper” argumentation leading to firm decisions.

Although a comparison of the number of different constructs employed to map the argumentation in each of the workshops is not particularly meaningful, we can examine the relative use of constructs within each workshop:

- During the CP workshop, an average of 3.17 positions were proposed in response to each issue; during the LSP workshop, there were 9.14 positions per issue, and during the DT workshop, 11 positions for every issue proposed.
- During the CP workshop, each position gave rise to an average of 1.27 arguments for or against that position; during the LSP workshop, the value was 3.56 arguments per position; and during the DT workshop, there were an average of 1.77 arguments for each position.
- The ratio of pro to con argumentation during the CP workshop was 1.15, while during the LSP workshop this ratio was 4.67. This ratio cannot be calculated for the DT workshop, where the analysis somewhat surprisingly revealed a complete absence of con argumentation.

These comparisons suggest that the CP methodology tends to lead to fewer positions (answers) being proposed for each issue raised (question posed), and that those positions in turn give rise to less argumentation, but that CP has a greater propensity for balanced argumentation, in terms of ratio of pro and con arguments put forward. This is not entirely surprising, since CP explicitly encourages positions and assumptions to be challenged by the group, while LSP–especially as delivered online–tends towards an exploration of individual values, which would often then be supported by other participants. The DT workshop seems effective in eliciting positions, but the absence of con argumentation is puzzling until one remembers, once again, that designers who otherwise might be expected to participate were not present in this case.

## 4 Thematic Engagement in the RD Workshop

The RD group was tasked with trying to take the outputs of the three user workshops forward, towards clarifying a problem domain and a potential robotic solution which could be achieved within the next 5 years. It was not expected that they would actually be successful in this task; rather, the purpose was to analyze how they chose to engage with the information provided and with each other in the process of decision-making when neither problem nor user group had been pre-defined. The thematic map was used as a prompt to help visualise the points raised and ideate preliminary concepts. It was also used to seek elaboration on user insights as the quotes from which each theme was derived were embedded in the interactive form of the map (which was manipulated by a co-facilitator and screen-shared for discussion). Unsurprisingly, when engaging with the map, RD tended to prioritise *Qualities of a Useful Robot,* in particular the “functional characteristics” whose first-order themes were “monitors me”, “alleviates the burden of management” and “help with physical tasks”. In this sense the LSP workshop provided the majority of the themes with which RD wanted to engage.

As expected, however, the majority of the tasks envisioned for the robots were well beyond the present capacities of the field, although many of the non-functional characteristics (reliable, efficient, fast, strong, transparent, etc.) are already within present scope.

All expressed the opinion that it was unsatisfactory not to be able to question the users directly, even if the oracle panel could answer all the questions posed. For the designers, this was particularly frustrating; some queried the value of receiving these insights via the research team as it did not fit with the model of innovation which they knew:

*“It has to be iterative, we have to go back, we have to have that dialogue to continue to refine our understanding of the problem and the users’, you know, ambitions to what this technology could be. To find something that matches those needs but that is also feasible within the context of our current technological abilities and the costs and all the other issues. …And then once we’ve come to some consensus that, yes, there is a problem here we can solve and it’s something that is valuable to those users, then we can come up with the design and then we can look at doing, kind of, a market analysis and is this something that’s feasible to build on a large scale.”* (RD/J)

Some RD did, however, acknowledge that such an approach inevitably narrows the perceived user population down to just the people they are able to engage with. As none were themselves disabled, in lieu of users, RD often drew on stories of disabled people they knew or assumptions about disability experience. However, they did demonstrate awareness of, and experience with, existing *non*-robotic care solutions as well as care robotics developments.

With regard to the content analysis, the term *Robot* was the most frequently used keyword in the discussion, with 88 instances; mean and median keyword frequency (8.11 and 3 instances respectively) indicates most keywords appeared far less frequently in the transcript (see Appendix 2 for frequency count). Instances of keywords occurring in the transcript were examined for their relevance to the themes they were drawn from, and matches counted. Direct matches (e.g., Keyword *Useful* in the phrase *quality of a useful robot*) were recorded for this analysis; partial or thematic matches [e.g (a robot that also benefits the general population) is *still a useful device to have*] are reserved for future discussion. Instances where multiple keywords appear in the same phrase (e.g., “*connects*” *to the “emergency” “services”*) are counted as one instance of reference to an IIRD theme. Table 5 identifies the IIRD themes referred to in the transcript and the frequency these were mentioned.

**TABLE 5:** IIRD themes mentioned during RD discussion and their frequency of use.

Themes in the map	From session			Instances
Physical cognitive impacts	—	LSP	—	7
Adaptation	—	—	DT	1
Mass market tech	CP	LSP	DT	2
Existing assistive tech	CP	LSP	DT	6
**Easy effortless**	—	LSP	—	1
**possible advantages of robots**	CP	—	—	1
Only produced through human interaction	CP	LSP	—	1
**QUALITIES OF A USEFUL ROBOT**	CP	LSP	DT	1
Intuitive and available but not intrusive	—	LSP	—	1
**Non-functional characteristics**	CP	LSP	—	2
Portable mobile unobtrusive	—	LSP	—	1
Voice activation	—	LSP	—	7
Sensors hearing, touch, vision, smell	—	LSP	—	1
Ability to navigate	CP	LSP	—	2
Strength	CP		—	1
Adaptable to needs and changing circumstances of user	CP	LSP	—	2
Connects to emergency services	—	LSP	—	2
**Interactive capacities**	CP	LSP	—	2
Living with disability	CP	LSP	—	3
Give a sense of companionship	CP	—	—	7
User co-created	—	LSP	—	5
**Functional characteristics**	CP	LSP	DT	4
Monitors me	—	LSP	DT	7
Alleviates the burden of management	—	LSP	DT	6
Help with physical tasks	CP	LSP	DT	16
Stretched resources	—	LSP	—	1
May be inevitable to maintain quality of life	—	LSP	—	1
Replacing humans	CP	—	—	1
Robots cannot care	CP	—	—	2
**(Tensions) in managing disability**	—	—	DT	4
Solutions are not always desirable	—	—	DT	1

Nb. Themes in bold are drawn from mid-levels of the IIRD, map.

Nb. Themes in BOLD, CAPS, are drawn from top levels of the IIRD, map.

The variation in the themes discussed and the frequency with which each was discussed can be used to highlight differences between the focus groups’ generation of useful topics for the RD group. Given that 1) the non-parametric variation in the frequency counts for use of themes, and 2) the themes were not necessarily independently generated in focus groups (a theme may have been raised by more than one of these groups), a Friedman test is used to compare between the focus groups’ generation of useful themes. The test indicates that there was a significant difference between the initial workshops in their association with IIRD themes raised in the RD workshop χ^2^ (2) = 9.77, *p* = 0.008. Post-hoc tests using Wilcoxon signed-rank test with Bonferroni-adjusted alpha of 0.017 indicate that the LSP workshop is associated with significantly more IIRD themes used in the RD workshop than the DT workshop (T = 160, Z = -2.98, *p* = 0.003). While this difference is recognised, Table 6 highlights that, given the relative number of themes each workshop is associated with, differences may be a product of the high number of themes generated by LSP.

**TABLE 6:** IIRD Themes mentioned during RD discussion as percentage of themes generated from first sessions.

	CP	LSP	DT
Total Themes Attached	54	65	29
Themes Mentioned in R&D	16	23	10
% Themes Mentioned in R&D	29.63	35.38	34.48
Cumulative Use in R&D	53	81	48

Common themes across the LSP, CP and DT workshops, such as the robot’s functional characteristics, the use of existing technology and the assistance with physical tasks were discussed on multiple occasions throughout the RD discussion. Individual workshops also contributed unique themes that were discussed on multiple occasions–of note, LSP not only generated more unique themes than the other workshops, but also more themes that were revisited by the RD group on multiple occasions.

## 5 Discussion

Overall, “care” emerges in all four workshops as a human quality produced through interaction between caregiver and cared-for. The themes in *Aspects of Care* thus aligned quite strongly with the definition put forward by Tronto (1993, as interpreted by Puig de la Bellacasa, 2017 p. 44) as comprising “the affective and ethical dispositions involved in concern, worry and taking responsibility for other’s well-being”. This, it was generally agreed, a robot could not and should not be expected to do.

As hoped, participants in the user workshops did commonly situate the discussions within their wider lived experiences in all methods deployed, although *Qualities of a useful robot* showed a range of sometimes contradictory themes, illuminating significantly different expectations and understandings of “useful”. As participants in the CP and LSP group were largely discussing robots they imagined as mobile and semi-autonomous, these two groups also stressed the need for the robot to be interactive, although they did not always discuss this using the same themes. This was markedly different from the DT group, who settled very early on an app as being sufficient to provide the functions they required.

In *Conceptions of Living,* the LSP and DT workshops elicited more focused reflections on individual needs and experiences, particularly the extra mental and physical energy that self-management of a variable disability often requires, noting these were concerns many people with disabilities share. For CP, management concerns were more aligned to the experience of *being cared for*, reflected in *Aspects of Care*.

Themes in the *Tensions* dimension represented the multifaceted nature of both robots and the experience of living with disability, pointing to the centrality of human experience in informing perceptions of the phenomenon in question (Boylorn, 2008) and the need to resist the decontextualisation of care practices often found in care robotics research (Maibaum et al., 2021). We used this dimension to capture dissonances and acknowledge potential irreconcilability, for example between those who envisioned their robot as a kind of friend and those who wanted their robot to perform its tasks and then place itself out of the way. As such, tensions “with robots” showed that respondents could envision robots being usefully incorporated into their lives, but also revealed a number of concerns which could dissipate the advantages robots could offer.

Critique of the thematic mapping during the RD group highlighted some of the challenges of interpretation and reaching common understanding in interdisciplinary research endeavours (Huutoniemi et al., 2010). However—given the epistemological and methodological differences between social science and engineering—it also shows the importance of using insights from one discipline to inform another (Stember, 1991; Payne, 1999). Our findings support the idea that care robots cannot be considered in isolation from the broader context of living with disability. Direct access to the end-user, as the designers in the RD group noted, is necessary to understand both context and problem-framing.

The research also points to a major misalignment between user expectations of “a useful robot” and those of RD, which, as Bradwell et al. (2019) found, tend to imagine the end user as more passive than users imagine themselves. For the most part, users wanted robots which could help manage the day-to-day details of living with disability so they could get on with the rest of their lives; although the protocols asked about specific tasks, they were often less engaged with what the robot could do as in how it would make them feel.

As care robotics development by definition targets vulnerable populations, the workshops confirmed that gaining early insight into user responses can highlight the sensitive and deeply personal context of care, and anxiety about the consequences of giving some aspects of this over to automation (McLeay et al., 2021). However, given the heterogeneity of these experiences even within the confines of our small sample, it is clear that finding ways for robotics designers to gain insights from a broad range of potential users is difficult. While the different methodologies employed in the workshops are all being used to explore the same problem space, they are doing so in subtly different ways which offer different things to an early-stage participatory design process. As the thematic mapping showed, CP lends itself well to an exploration of care as a value-laden, emotive and interpersonal experience. The hands-on narrative aspect of LSP elicits a fuller contextual response, allowing participants to situate a robot of their own design within their own daily life and use this to determine which principles they find important. This approach generated most of the themes used by the RD team, suggesting they were the most accessible to those trained in engineering or design. As the more standard approach, DT provides a set of well-defined and familiar steps to translate insights about a potential user-group into principles more familiar to designers, however, its particular structure meant that overall, it generated less thematic insight and therefore less opportunity for engagement by the RD team.

Overall, the three user workshops elicited a synthesised view on the lived experience of disability, experiences of care, expected characteristics of a desirable care robot, and tensions in each of these categories of consideration. Obtaining these broader viewpoints confirmed the need to consider how to develop and evaluate robot initiatives within the frameworks of existing care ecosystems (Van Aerschot and Parviainen, 2020). Indeed, many of the tensions identified by the participants are insoluble and will to a large extent depend upon both the context of deployment and the individual preferences of the end-user, more than the robot itself. Above all, however, the workshops highlighted the danger of automation reducing the cared-for person to an object to be acted upon by a machine, a “surface to be wiped down” or a problem to be solved.

Our findings underscore that user expectations of care robots should not be considered in isolation from the broader context of their lived experiences. Given the heterogeneity of these experiences, even within the confines of our small sample, it is clear that finding ways for robotics designers to gain insights from a broader range of users at the earliest stage of development will be key to shaping the field.

## 6 Limitations and Future Research Directions

Given the limited sample, it is not possible to generalise the thematic output as representative; likewise, although the protocols can be replicated, different facilitators and participants might produce significantly different outcomes. Further challenges were imposed by the need to move the workshops online due to COVID-19. Both CP and LSP translated well to the Zoom environment, however, DT presented a significant technical challenge for some participants, who had trouble using the Mural board despite having accomplished this in the pre-call training. This likely limited the range of creativity, and perhaps contributed to the shaping of solutions towards apps, as these were familiar technologies everyone knew how to use. However, there were also significant benefits to allowing disabled people to participate from the comfort and certainty of their own home, avoiding the physical cost and effort of travel which suggests exploring mitigation options such as using a different platform or recording technique would be fruitful, as future research using these methodologies in sequence to develop an actual robotics project is envisioned. It is also clear these efforts would benefit from deeper engagement with services research in order to understand how the embedding of new artefacts will be challenged by existing systems.

## 7 Conclusion

Our research confirms that there is potential in bringing roboticists and designers into contact with the user group at an early stage of the process, although perhaps not at the initial ideation stages exemplified by the CP and LSP groups. Here, there may be more value in allowing potential users to first explore their lived experiences and their hopes and anxieties about the introduction of robots into their lives without specifying technical limitations. Used in sequence, however, the three methods could provide both early insight and a dedicated user-group, with a “visioning phase” consisting of an initial CP session to explore ideas, followed by an LSP session to develop scenarios and situations in which a robot might be beneficially deployed for technologically feasible tasks. These could be used to develop the persona and problem context for a DT workshop which then incorporates the RD team and other stakeholders to explore the solution space together, thus focussing on those options which have a chance of being developed.

The mismatch of user and RD requirements revealed by these workshops highlight that it is crucial to persevere in finding ways for social and engineering sciences to better complement each other. Methods which can translate esoteric values into engineering principles may both improve the meaningful incorporation of users earlier in the process, and embed a fuller range of social sciences (beyond design and psychology) into robotics research. The co-creative methodology presented here represents a new direction for inclusive engagement from the start.

## Data Availability

The datasets presented in this article are not readily available because the participants have not consented to the sharing of recordings or transcripted data. Requests to access the quantitative or IBIS datasets should be directed to the lead author.

## Footnotes

1 For example, government spending on home care services in the UK fell by 19.4% between 2010/11 and 2013/14 and has continued to drop since (Draper and Sorell, 2017).

2 The EU has been explicit about its desire to exploit this “Silver Economy” as a “driver of economic growth”. See https://digital-strategy.ec.europa.eu/en/library/growing-silver-economy-europe.

3 Probably best exemplified by Boston Dynamics: https://www.youtube.com/watch?v=fn3KWM1kuAw.

4 See, for example: https://www.theguardian.com/society/2020/sep/07/robots-used-uk-care-homes-help-reduce-loneliness.

5
https://www.malavida.com/en/soft/inspiration/.

6
https://cognexus.org/download_compendium.htm.

7
http://www.casos.cs.cmu.edu/projects/automap/AutoMap.
