# Supplementary material for: Improving Inclusivity in Robotics Design: An Exploration of Methods for Upstream Co-Creation
Source: Front Robot AI. 2022 Jun 21;9:731006. doi: 10.3389/frobt.2022.731006 (PMC9272459; doi:10.3389/frobt.2022.731006)
Supplement: Supplementary file 1 [file Table1.docx]

**Appendix 1**

**Table 7. Participant demographics for the focus-group workshops**

| Workshop | N | Age Range | Sex | Ethnicity | Self-reported disabilities* |
| --- | --- | --- | --- | --- | --- |
| Community Philosophy | 8 | 31 - 71 | 4F, 4M | 6 White 2 Black/African/ Caribbean/Black British | Deafness, partial blindness, partial paralysis, unilateral deafness, osteoarthritis, fibromyalgia, rheumatoid arthritis, spinal sclerosis |
| Lego  Serious  Play | 6 | 26 - 56 | 3F, 3M | 4 White  1 Mixed 1 Asian/Asian British | Rheumatoid arthritis, systemic lupus, multiple sclerosis, heart failure, autoimmune disorder, macular degeneration |
| Design Thinking | 6 | 33 - 74 | 2F, 4M | 4 White 2 Asian/Asian British | Partial paralysis, deafness, impaired-mobility, cerebral palsy, COPD, ulcerative colitis |

* Note that some people reported more than one.
