# Supplementary material for: Improving Inclusivity in Robotics Design: An Exploration of Methods for Upstream Co-Creation
Source: Front Robot AI. 2022 Jun 21;9:731006. doi: 10.3389/frobt.2022.731006 (PMC9272459; doi:10.3389/frobt.2022.731006)
Supplement: Supplementary file 2 [file Table2.docx]

**Appendix 2: List of terms aggregated using stemming and the list of keywords drawn from the IIRD map**

**Table 8: Stemmed Terms**

| **Original Term** | **Stemmed Term** | **Frequency in Text** |
| --- | --- | --- |
| **going** | **go** | **45** |
| **having** | **have** | **23** |
| **doing** | **do** | **20** |
| **looking** | **look** | **20** |
| **users** | **user** | **18** |
| **robotics** | **robot** | **17** |
| **tasks** | **task** | **17** |
| **hands** | **hand** | **16** |
| **robots** | **robot** | **16** |
| **talking** | **talk** | **14** |
| **trying** | **try** | **14** |
| **used** | **use** | **13** |
| **robotic** | **robot** | **12** |
| **getting** | **get** | **10** |
| **potentially** | **potential** | **10** |
| **asked** | **ask** | **9** |
| **does** | **do** | **9** |
| **mentioned** | **mention** | **9** |
| **designed** | **design** | **8** |
| **devices** | **device** | **8** |
| **cupboards** | **cupboard** | **7** |
| **earlier** | **early** | **7** |
| **lots** | **lot** | **7** |
| **others** | **other** | **7** |
| **problems** | **problem** | **7** |
| **talked** | **talk** | **7** |
| **wanted** | **want** | **7** |
| **based** | **base** | **6** |
| **carer** | **care** | **6** |
| **constraints** | **constraint** | **6** |
| **ordering** | **order** | **6** |
| **products** | **product** | **6** |
| **worked** | **work** | **6** |
| **appropriately** | **appropriate** | **5** |
| **challenges** | **challenge** | **5** |
| **designers** | **designer** | **5** |
| **disabilities** | **disability** | **5** |
| **functionality** | **functional** | **5** |
| **issues** | **issue** | **5** |
| **ones** | **one** | **5** |
| **reducing** | **reduce** | **5** |
| **systems** | **system** | **5** |
| **wondering** | **wonder** | **5** |
| **activities** | **activity** | **4** |
| **alleviates** | **alleviate** | **4** |
| **becomes** | **become** | **4** |
| **comes** | **come** | **4** |
| **delivered** | **deliver** | **4** |
| **easier** | **easy** | **4** |
| **feels** | **feel** | **4** |
| **fits** | **fit** | **4** |
| **goals** | **goal** | **4** |
| **helps** | **help** | **4** |
| **ideas** | **idea** | **4** |
| **identifying** | **identify** | **4** |
| **iterative** | **iterate** | **4** |
| **jars** | **jar** | **4** |
| **meals** | **meal** | **4** |
| **monitoring** | **monitor** | **4** |
| **projects** | **project** | **4** |
| **says** | **say** | **4** |
| **seems** | **seem** | **4** |
| **solutions** | **solution** | **4** |
| **sounds** | **sound** | **4** |
| **totally** | **total** | **4** |
| **wearable** | **wear** | **4** |
| **achievable** | **achieve** | **3** |
| **areas** | **area** | **3** |
| **bits** | **bit** | **3** |
| **cameras** | **camera** | **3** |
| **carers** | **care** | **3** |
| **changed** | **change** | **3** |
| **committing** | **commit** | **3** |
| **customers** | **customer** | **3** |
| **depends** | **depend** | **3** |
| **exists** | **exist** | **3** |
| **feeding** | **feed** | **3** |
| **homes** | **home** | **3** |
| **hours** | **hour** | **3** |
| **interactive** | **interact** | **3** |
| **legs** | **leg** | **3** |
| **manipulator** | **manipulate** | **3** |
| **minds** | **mind** | **3** |
| **mounted** | **mount** | **3** |
| **moved** | **move** | **3** |
| **putting** | **putt** | **3** |
| **reminders** | **reminder** | **3** |
| **requirements** | **requirement** | **3** |
| **sensors** | **sensor** | **3** |
| **services** | **service** | **3** |
| **shopping** | **shop** | **3** |
| **solved** | **solve** | **3** |
| **stakeholders** | **stakeholder** | **3** |
| **starting** | **start** | **3** |
| **struggling** | **struggle** | **3** |
| **takes** | **take** | **3** |
| **technologies** | **technology** | **3** |
| **types** | **type** | **3** |
| **advantages** | **advantage** | **2** |
| **animals** | **animal** | **2** |
| **answers** | **answer** | **2** |
| **asking** | **ask** | **2** |
| **aspects** | **aspect** | **2** |
| **assumptions** | **assumption** | **2** |
| **avengers** | **avenge** | **2** |
| **biases** | **bias** | **2** |
| **bigger** | **big** | **2** |
| **called** | **call** | **2** |
| **changing** | **change** | **2** |
| **characteristics** | **characteristic** | **2** |
| **cleaning** | **clean** | **2** |
| **combined** | **combine** | **2** |
| **commands** | **command** | **2** |
| **comments** | **comment** | **2** |
| **committees** | **committee** | **2** |
| **compared** | **compare** | **2** |
| **concerns** | **concern** | **2** |
| **defined** | **define** | **2** |
| **defining** | **define** | **2** |
| **developing** | **develop** | **2** |
| **differently** | **different** | **2** |
| **discussions** | **discussion** | **2** |
| **doable** | **do** | **2** |
| **exoskeletons** | **exoskeleton** | **2** |
| **expecting** | **expect** | **2** |
| **explicitly** | **explicit** | **2** |
| **genuinely** | **genuine** | **2** |
| **gets** | **get** | **2** |
| **gives** | **give** | **2** |
| **groups** | **group** | **2** |
| **happens** | **happen** | **2** |
| **headings** | **heading** | **2** |
| **identified** | **identify** | **2** |
| **impairment** | **impair** | **2** |
| **interfaces** | **interface** | **2** |
| **items** | **item** | **2** |
| **jeeves** | **jeeve** | **2** |
| **knows** | **know** | **2** |
| **limitations** | **limitation** | **2** |
| **managing** | **manage** | **2** |
| **miles** | **mile** | **2** |
| **monitors** | **monitor** | **2** |
| **months** | **month** | **2** |
| **nailed** | **nail** | **2** |
| **nappies** | **nappy** | **2** |
| **noticed** | **notice** | **2** |
| **participants** | **participant** | **2** |
| **parties** | **party** | **2** |
| **pauses** | **pause** | **2** |
| **pilots** | **pilot** | **2** |
| **questions** | **question** | **2** |
| **reaching** | **reach** | **2** |
| **requires** | **require** | **2** |
| **stories** | **story** | **2** |
| **suffered** | **suffer** | **2** |
| **targeted** | **target** | **2** |
| **views** | **view** | **2** |
| **accessing** | **access** | **1** |
| **acknowledging** | **acknowledge** | **1** |
| **activation** | **activate** | **1** |
| **actively** | **active** | **1** |
| **adapted** | **adapt** | **1** |
| **adding** | **add** | **1** |
| **adjusting** | **adjust** | **1** |
| **affordable** | **afford** | **1** |
| **aided** | **aid** | **1** |
| **allowing** | **allow** | **1** |
| **allows** | **allow** | **1** |
| **ambitions** | **ambition** | **1** |
| **answered** | **answer** | **1** |
| **appears** | **appear** | **1** |
| **appliances** | **appliance** | **1** |
| **apps** | **app** | **1** |
| **assessing** | **assess** | **1** |
| **assisted** | **assist** | **1** |
| **assistive** | **assist** | **1** |
| **associated** | **associate** | **1** |
| **assuming** | **assume** | **1** |
| **attached** | **attach** | **1** |
| **automated** | **automate** | **1** |
| **backs** | **back** | **1** |
| **balancing** | **balance** | **1** |
| **basing** | **base** | **1** |
| **beamed** | **beam** | **1** |
| **beams** | **beam** | **1** |
| **bills** | **bill** | **1** |
| **bodied** | **body** | **1** |
| **breaking** | **break** | **1** |
| **bringing** | **bring** | **1** |
| **buildings** | **building** | **1** |
| **capacities** | **capacity** | **1** |
| **cared** | **care** | **1** |
| **caring** | **care** | **1** |
| **cases** | **case** | **1** |
| **catches** | **catch** | **1** |
| **causing** | **cause** | **1** |
| **caveats** | **caveat** | **1** |
| **chopping** | **chop** | **1** |
| **chores** | **chore** | **1** |
| **closely** | **close** | **1** |
| **closer** | **close** | **1** |
| **clusters** | **cluster** | **1** |
| **cockpits** | **cockpit** | **1** |
| **colleagues** | **colleague** | **1** |
| **commercially** | **commercial** | **1** |
| **companies** | **company** | **1** |
| **components** | **component** | **1** |
| **conceived** | **conceive** | **1** |
| **conceptions** | **conception** | **1** |
| **concisely** | **concise** | **1** |
| **connects** | **connect** | **1** |
| **consulted** | **consult** | **1** |
| **contexts** | **context** | **1** |
| **continuously** | **continuous** | **1** |
| **controlling** | **control** | **1** |
| **countermanded** | **countermand** | **1** |
| **crashes** | **crash** | **1** |
| **created** | **create** | **1** |
| **culturally** | **cultural** | **1** |
| **cyclical** | **cyclic** | **1** |
| **daunting** | **daunt** | **1** |
| **debilitating** | **debilitate** | **1** |
| **decisions** | **decision** | **1** |
| **decorated** | **decorate** | **1** |
| **delivering** | **deliver** | **1** |
| **deployable** | **deploy** | **1** |
| **described** | **describe** | **1** |
| **describing** | **describe** | **1** |
| **descriptions** | **description** | **1** |
| **details** | **detail** | **1** |
| **developed** | **develop** | **1** |
| **difficulties** | **difficulty** | **1** |
| **disadvantages** | **disadvantage** | **1** |
| **discussed** | **discuss** | **1** |
| **discussing** | **discuss** | **1** |
| **disgustingly** | **disgusting** | **1** |
| **dispensing** | **dispense** | **1** |
| **distinguishably** | **distinguishable** | **1** |
| **dosages** | **dosage** | **1** |
| **doughnuts** | **doughnut** | **1** |
| **dressed** | **dress** | **1** |
| **eating** | **eat** | **1** |
| **embodied** | **embody** | **1** |
| **emergencies** | **emergency** | **1** |
| **emotionality** | **emotional** | **1** |
| **encouraging** | **encourage** | **1** |
| **ended** | **end** | **1** |
| **engineered** | **engineer** | **1** |
| **environmentally** | **environmental** | **1** |
| **envisaged** | **envisage** | **1** |
| **episodes** | **episode** | **1** |
| **examples** | **example** | **1** |
| **exercised** | **exercise** | **1** |
| **expected** | **expect** | **1** |
| **extending** | **extend** | **1** |
| **faces** | **face** | **1** |
| **failures** | **failure** | **1** |
| **falling** | **fall** | **1** |
| **feeds** | **feed** | **1** |
| **fewer** | **few** | **1** |
| **fields** | **field** | **1** |
| **fills** | **fill** | **1** |
| **filtering** | **filter** | **1** |
| **fixated** | **fixate** | **1** |
| **forms** | **form** | **1** |
| **framed** | **frame** | **1** |
| **frequently** | **frequent** | **1** |
| **functionalities** | **functional** | **1** |
| **funding** | **fund** | **1** |
| **furthered** | **further** | **1** |
| **goes** | **go** | **1** |
| **grandparents** | **grandparent** | **1** |
| **guaranteed** | **guarantee** | **1** |
| **gurus** | **guru** | **1** |
| **hazards** | **hazard** | **1** |
| **helper** | **help** | **1** |
| **helpfully** | **helpful** | **1** |
| **hobbies** | **hobby** | **1** |
| **houses** | **house** | **1** |
| **humans** | **human** | **1** |
| **impacts** | **impact** | **1** |
| **impaired** | **impair** | **1** |
| **implementation** | **implement** | **1** |
| **importantly** | **important** | **1** |
| **improves** | **improve** | **1** |
| **informing** | **inform** | **1** |
| **ingredients** | **ingredient** | **1** |
| **interacted** | **interact** | **1** |
| **interactions** | **interaction** | **1** |
| **interpreting** | **interpret** | **1** |
| **invested** | **invest** | **1** |
| **ipads** | **ipad** | **1** |
| **iwatches** | **iwatche** | **1** |
| **jumping** | **jump** | **1** |
| **kinds** | **kind** | **1** |
| **kitchens** | **kitchen** | **1** |
| **later** | **late** | **1** |
| **levels** | **level** | **1** |
| **limbs** | **limb** | **1** |
| **longer** | **long** | **1** |
| **losing** | **lose** | **1** |
| **makes** | **make** | **1** |
| **maps** | **map** | **1** |
| **matches** | **match** | **1** |
| **medicines** | **medicine** | **1** |
| **mentioning** | **mention** | **1** |
| **motivated** | **motivate** | **1** |
| **mouths** | **mouth** | **1** |
| **needing** | **need** | **1** |
| **nontechnical** | **nontechnic** | **1** |
| **novices** | **novice** | **1** |
| **opens** | **open** | **1** |
| **operating** | **operate** | **1** |
| **organising** | **organise** | **1** |
| **panels** | **panel** | **1** |
| **personalised** | **personalise** | **1** |
| **photos** | **photo** | **1** |
| **picking** | **pick** | **1** |
| **planning** | **plan** | **1** |
| **powered** | **power** | **1** |
| **predicting** | **predict** | **1** |
| **predictive** | **predict** | **1** |
| **preparing** | **prepare** | **1** |
| **presented** | **present** | **1** |
| **processor** | **process** | **1** |
| **produced** | **produce** | **1** |
| **productization** | **product** | **1** |
| **promoting** | **promote** | **1** |
| **prompted** | **prompt** | **1** |
| **prompts** | **prompt** | **1** |
| **proposals** | **proposal** | **1** |
| **qualities** | **quality** | **1** |
| **raising** | **raise** | **1** |
| **ranging** | **range** | **1** |
| **recipients** | **recipient** | **1** |
| **redesigned** | **redesigne** | **1** |
| **relationships** | **relationship** | **1** |
| **remembering** | **remember** | **1** |
| **reminded** | **remind** | **1** |
| **reminding** | **remind** | **1** |
| **remodelling** | **remodel** | **1** |
| **replacing** | **replace** | **1** |
| **required** | **require** | **1** |
| **responds** | **respond** | **1** |
| **responsivity** | **responsive** | **1** |
| **restrictions** | **restriction** | **1** |
| **retrieves** | **retrieve** | **1** |
| **retrieving** | **retrieve** | **1** |
| **roles** | **role** | **1** |
| **rolled** | **roll** | **1** |
| **rules** | **rule** | **1** |
| **scanned** | **scan** | **1** |
| **scottish** | **scotland** | **1** |
| **sections** | **section** | **1** |
| **selling** | **sell** | **1** |
| **shaking** | **shake** | **1** |
| **shaping** | **shape** | **1** |
| **shared** | **share** | **1** |
| **shoes** | **shoe** | **1** |
| **skills** | **skill** | **1** |
| **smaller** | **small** | **1** |
| **sorts** | **sort** | **1** |
| **sounded** | **sound** | **1** |
| **stages** | **stage** | **1** |
| **standards** | **standard** | **1** |
| **stands** | **stand** | **1** |
| **statements** | **statement** | **1** |
| **stations** | **station** | **1** |
| **storing** | **store** | **1** |
| **stretched** | **stretch** | **1** |
| **sufficiently** | **sufficient** | **1** |
| **suggesting** | **suggest** | **1** |
| **surfaces** | **surface** | **1** |
| **suspecting** | **suspect** | **1** |
| **tackling** | **tackle** | **1** |
| **tellies** | **telly** | **1** |
| **tensions** | **tension** | **1** |
| **themes** | **theme** | **1** |
| **thoughts** | **thought** | **1** |
| **throwing** | **throw** | **1** |
| **timelines** | **timeline** | **1** |
| **tipping** | **tip** | **1** |
| **tried** | **try** | **1** |
| **trips** | **trip** | **1** |
| **twisting** | **twist** | **1** |
| **uncertainties** | **uncertainty** | **1** |
| **understands** | **understand** | **1** |
| **unloads** | **unload** | **1** |
| **using** | **use** | **1** |
| **values** | **value** | **1** |
| **vectors** | **vector** | **1** |
| **vegetables** | **vegetable** | **1** |
| **visions** | **vision** | **1** |
| **warehousing** | **warehouse** | **1** |
| **wearables** | **wear** | **1** |
| **wheelchairs** | **wheelchair** | **1** |
| **workers** | **worker** | **1** |
| **workshopping** | **workshop** | **1** |
| **receiving** | **receive** | **1** |
| **Living** | **Live** | **1** |

**Table 8: Keywords from IIRD Map used by RD group**

| **Keywords from IIRD Themes** | **Frequency in R&D Transcript** |
| --- | --- |
| **ROBOT** | **88** |
| **need** | **36** |
| **user** | **36** |
| **help** | **33** |
| **other** | **31** |
| **look** | **30** |
| **different** | **29** |
| **task** | **28** |
| **Physical** | **25** |
| **make** | **24** |
| **change** | **21** |
| **CARE** | **21** |
| **work** | **20** |
| **tech** | **18** |
| **down** | **18** |
| **needs** | **18** |
| **good** | **17** |
| **general** | **15** |
| **possible** | **14** |
| **disability** | **14** |
| **away** | **12** |
| **first** | **11** |
| **USEFUL** | **11** |
| **functional** | **11** |
| **voice** | **10** |
| **difficult** | **9** |
| **feel** | **9** |
| **day** | **8** |
| **question** | **8** |
| **life** | **8** |
| **appropriate** | **8** |
| **over** | **8** |
| **monitor** | **7** |
| **management** | **7** |
| **interact** | **7** |
| **constraint** | **7** |
| **easy** | **7** |
| **always** | **6** |
| **solution** | **6** |
| **human** | **6** |
| **exist** | **5** |
| **support** | **5** |
| **part** | **5** |
| **navigate** | **5** |
| **interaction** | **5** |
| **burden** | **5** |
| **companionship** | **4** |
| **cognitive** | **4** |
| **bias** | **4** |
| **alleviate** | **4** |
| **impact** | **4** |
| **big** | **3** |
| **vision** | **3** |
| **strength** | **3** |
| **service** | **3** |
| **sensor** | **3** |
| **mobile** | **3** |
| **everything** | **3** |
| **assist** | **3** |
| **aspect** | **3** |
| **advantage** | **3** |
| **ability** | **3** |
| **characteristic** | **3** |
| **manage** | **3** |
| **practical** | **3** |
| **quality** | **3** |
| **value** | **2** |
| **taken** | **2** |
| **Social** | **2** |
| **sense** | **2** |
| **replace** | **2** |
| **remove** | **2** |
| **profile** | **2** |
| **nonfunctional** | **2** |
| **market** | **2** |
| **machine** | **2** |
| **learn** | **2** |
| **individual** | **2** |
| **development** | **2** |
| **connect** | **2** |
| **break** | **2** |
| **allow** | **2** |
| **Adaptation** | **2** |
| **Control** | **2** |
| **decision** | **2** |
| **emergency** | **2** |
| **genuine** | **2** |
| **personal** | **2** |
| **touch** | **1** |
| **respond** | **1** |
| **reliable** | **1** |
| **receive** | **1** |
| **points** | **1** |
| **maintain** | **1** |
| **love** | **1** |
| **intrusive** | **1** |
| **interests** | **1** |
| **hearing** | **1** |
| **circumstances** | **1** |
| **available** | **1** |
| **resources** | **1** |
| **LIVE** | **1** |
| **mass** | **1** |
